# Homotherapy for heteropathy of chronic kidney disease and oligoasthenozoospermia through regulating SIRT1/NF-κB pathway by Shenqi pills

**DOI:** 10.3389/fphar.2025.1551423

**Published:** 2025-06-09

**Authors:** Shuo Huang, Qihan Luo, Xinyue Li, Yiming Liu, Jiale Wei, Sichen Wang, Ping Qiu, Changyu Li

**Affiliations:** ^1^ School of Pharmaceutical Sciences, Zhejiang Chinese Medical University, Hangzhou, China; ^2^ School of Basic Medical Sciences, Zhejiang Chinese Medical University, Hangzhou, China; ^3^ Academy of Chinese Medical Sciences, Zhejiang Chinese Medical University, Hangzhou, China

**Keywords:** shenqi pill, homotherapy for heteropathy, SIRT1/NF-κB pathway, chronic kidney disease, oligoasthenospermia

## Abstract

**Background:**

Chronic kidney disease (CKD), defined by a glomerular filtration rate (GFR) below 60 mL/min/1.73 m^2^ for over 3 months, is a significant global health concern, often progressing to end-stage renal disease (ESRD). Oligoasthenospermia (OA), characterized by reduced sperm count or quality, affects male fertility, contributing to infertility in approximately 15% of couples worldwide. Both conditions share features of yang deficiency, including fatigue, cold intolerance, and weakness. Shenqi Pill (SQP), a Traditional Chinese Medicine (TCM) formula, replenishes kidney yang and demonstrates efficacy in treating yang deficiency-related diseases such as CKD and OA. However, the molecular mechanisms underlying its therapeutic effects remain unclear.

**Methods:**

This study combined ultra-performance liquid chromatography-quadrupole time-of-flight mass spectrometry (UPLC-Q-TOF/MS), network pharmacology, and machine learning to identify SQP’s active compounds and potential targets. A CKD model was induced in C57BL/6 mice via adenine administration, followed by SQP treatment (0.8 or 1.6 g/kg/day) for 50 days. Renal function, histopathology, and molecular pathways were evaluated. Additionally, *in vitro* assays were performed to validate SQP’s effects on OA using GC-1spg spermatogonia.

**Results:**

41 compounds in SQP were identified. Network pharmacology suggested SQP ameliorates CKD and OA by modulating cellular senescence, with SIRT1, RELA, and NFKB1 as key targets. *In vivo*, SQP improved renal dysfunction, reduced glomerular atrophy, tubular dilation, and collagen deposition, with higher doses demonstrating superior efficacy. RNA-Seq analysis highlighted SQP’s regulation of the SIRT1/NF-κB pathway and cellular senescence. ELISA, β-galactosidase staining, and Western blotting confirmed reduced senescence-associated secretory phenotype (SASP) release and normalization of SIRT1/NF-κB1 activity. *In vitro*, SQP-containing serum alleviated cellular senescence in GC-1spg spermatogonia by mitigating SIRT1/NF-κB1 disruptions without cytotoxicity.

**Conclusion:**

SQP demonstrates therapeutic potential for CKD and OA by targeting the SIRT1/NF-κB signaling pathway, providing evidence for its clinical application in treating kidney-yang deficiency-related diseases.

## Graphical Abstract

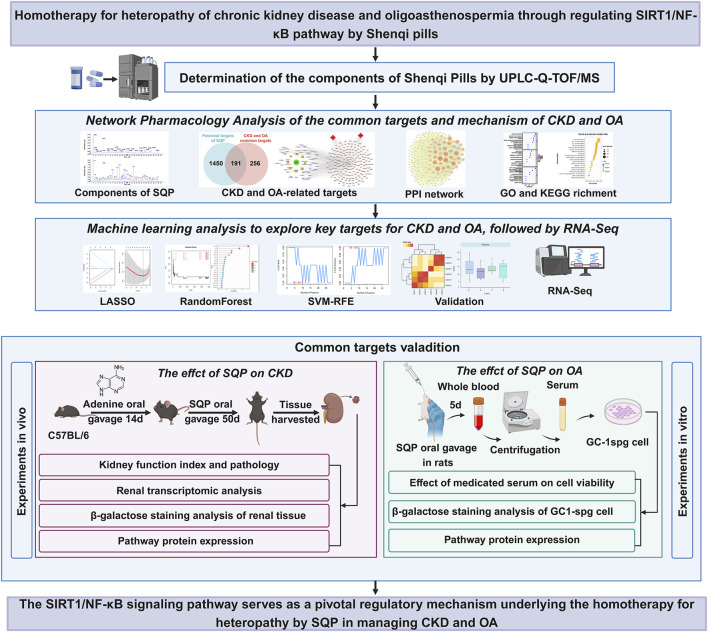

## 1 Introduction

Chronic kidney disease (CKD) refers to a pathological state characterized by a glomerular filtration rate (GFR) below 60 mL/min/1.73 m^2^ of body surface area, persisting for more than 3 months due to various etiologies (Chinese Preventive Medicine Association for Kidney, 2023). Patients with CKD often exhibit increased renal inflammation, tubular atrophy, tubulointerstitial fibrosis, and glomerulosclerosis, potentially progressing to renal failure or end-stage renal disease (ESRD) (Wang et al., 2022). CKD has emerged as a significant global public health issue and a key contributor to the increasing burden of non-communicable diseases (Jha et al., 2012).

Oligoasthenospermia (OA) is a condition where a male partner has a reduced number of normal sperm or compromised sperm quality, leading to the inability of the female partner to conceive for over a year (Ferlin et al., 2021). Current data indicate that infertility affects approximately 15% (approximately 50 million) of couples of reproductive ages worldwide, with male infertility accounting for up to 50% of these cases (Lu et al., 2020). Evidence suggests that male semen quality declines annually and the impact of male infertility on the next-generation is a matter of global concern (Levine et al., 2023).

In recent years, substantial evidence has demonstrated that traditional Chinese medicine (TCM) has a significant therapeutic efficacy and a good safety profile. TCM has been widely used in clinical settings to improve renal function and enhance the quality of male reproductive cells (Zhao et al., 2022; Wang et al., 2023). Clinical studies have revealed that, upon reaching a certain disease stage, patients with CKD or OA exhibit symptoms of yang deficiency, such as cold limbs, aversion to cold, fatigue, and general weakness, suggesting a possible common pathological mechanism. The TCM theory posits that kidney yang is the fundamental source of yang qi in the body, playing a crucial role in stimulating and warming various organs and tissues. Therefore, treatment aimed at replenishing kidney yang may be key to alleviating these two diseases (Wang et al., 2022; Gao et al., 2023). Shenqi pill (SQP) is a representative formula used in TCM for warming and tonifying kidney yang. It was first recorded in the “Jin Gui Yao Lue” and is also known as the Ba Wei Shenqi pill or the Gui Fu Di Huang pill. Eight herbal constituents make up the majority of the formula: *Cinnamomi cortex* (Rougui), *Aconiti lateralis radix praeparata* (Fuzi), *Rehmanniae radix* (Dihuang), *Corni fructus* (Shanzhuyu), *Moutan cortex* (Mudanpi), *Dioscoreae rhizoma* (Shanyao), *Poria* (Fuling)*,* and *Alisma rhizoma* (Zexie) (Zhou et al., 2016; Lin et al., 2023). All the plant names have been checked with theplantlist datasets (http://www.theplantlist.org). SQP has demonstrated significant efficacy in treating diseases associated with kidney yang deficiency (Nan et al., 2016). The clinical applications of SQP have expanded with ongoing research, with reports demonstrating beneficial effects on CKD and OA (Gao et al., 2014; Liu Y. et al., 2024). However, the molecular mechanisms underlying the “homotherapy for heteropathy” approach using SQP remain scarcely reported.

Network pharmacology provides a distinct viewpoint on the way medications treat illnesses, which is consistent with TCM’s emphasis on the whole body and its fundamental concept of “multi-components, multi-targets, and multi-pathways.” Both approaches have studied this issue from an interconnected viewpoint (Luo et al., 2022; Liu Y. et al., 2024). This study aimed to combine ultra-performance liquid chromatography-quadrupole time-of-flight mass spectrometry (UPLC-Q-TOF/MS) technology, network pharmacology, and *in vivo* and *in vitro* experiments to predict the potential common targets and synergistic mechanisms regulating CKD and OA. This study establishes a scientific foundation for medical research and the discovery of novel TCM medications for the treatment of these two diseases.

## 2 Methods

### 2.1 Detection of active components in SQP using UPLC-Q-TOF/MS

Sample preparation: The aqueous extract from SQP was obtained utilizing a solid-phase extraction column. The processing conditions involved a 3 mL water rinse, followed by elution with 3 mL of 95% methanol.

Chromatographic conditions: An ACQUITY UPLC BEH C18 column (100 × 2.1 mm, 1.7 μm) was used. The mobile phase in this experiment was composed of acetonitrile (A) and 0.1% formic acid in water (B), and a gradient elution procedure was used, as shown in Supplementary Table S1. The flow rate of the solution was set at 0.3 mL/min, with the sample tray temperature maintained at 8°C, the column temperature controlled at 40°C, and an injection volume of 5 μL.

Mass spectrometry conditions: A TurboIonSpray ion source with electrospray ionization (ESI) time-of-flight mass spectrometer was used in both the positive and negative ion scanning modes. Tandem mass spectrometry data were acquired using the information-dependent acquisition (IDA) technique in high-sensitivity mode. The declustering potential (DP) was adjusted at ± 60 V for both positive and negative ion modes, with a collision energy of 35 ± 15 eV. The IDA parameters included eliminating isotopes of less than 4 Da and monitoring 12 candidate ions per cycle.

Compound identification: Compounds were identified using the SCIEX OS software’s built-in TCM secondary database, the TCM MS/MS library, which included primary accurate mass, isotopic distribution ratio, and secondary fragment MS/MS data. This was extended using a self-built library that included all SQP components.

### 2.2 Network pharmacology study on the “homeotherapy for heteropathy” of SQP for CKD and OA

By reviewing the literature and integrating the results of UPLC-Q-TOF/MS, the components of SQP after supplementation were identified for further analysis. The predicted targets of the aqueous extract components of SQP were collected using the TCMSP, BATMAN-TCM, PharmMapper, HERB, SEA, and STITCH databases. Uniprot was used to map the target protein names to their corresponding gene names. Disease target information was gathered using “chronic kidney diseases” and “oligoasthenospermia” as keywords in databases including CTD, DisGenet, GeneClip3, GeneCards, IPA, MalaCards, OMIM, TCMIP, and TTD. The median of the disease relevance score was used as a cutoff to filter the targets, and the high-frequecy targets in these databases were subsequently obtained by Upset R package. Predicted compounds and disease targets were integrated to indentify prospective targets associated with SQP’s homotherapy for heteropathy effects, which are represented by a Venn diagram. The intersecting targets were then loaded into Cytoscape 3.6.0 to create a drug-component-disease-target network that displayed component-target connections.

### 2.3 Selecting intersecting targets and building a protein-protein interaction network

The overlapped targets were uploaded to the STRING database, using the species “*Homo sapiens*” and the minimal interaction threshold “highest confidence (0.900). “Data from the protein-protein interaction (PPI) network model were subsequently entered into Cytoscape 3.6.0. In the graph, node size was positively correlated with the degree value, whereas edge color and thickness were positively correlated with betweenness centrality.

### 2.4 Functional enrichment analysis

The genes from the PPI network targets were uploaded to the DAVID database for GO and KEGG pathway enrichment analyses, with a P-value <0.05 set as the filtering criterion.

### 2.5 Application of machine learning for screening hub genes

The least absolute shrinkage and selection operator (LASSO) regression analysis is conducted using the “glmnet” package (Friedman et al., 2010). The RandomForest ensemble learning method is accomplished through the “randomForest” package. The support vector machine - recursive feature elimination (SVM-RFE) feature selection technique is implemented by combining the “e1071” and “caret” packages (Dimitriadou et al., 2006; Kuhn, 2008).

### 2.6 Experimental validation

#### 2.6.1 Animal

The Animal Experiment Research Center at Zhejiang Chinese Medical University provided 36 male C57BL/6 mice (specific-pathogen free grade (SPF), 8-weeks-old, 22 ± 2 g) and 18 male Sprague–Dawley (SD) rats (SPF, grade 250 ± 10 g). They were maintained under regulated conditions (temperature: 21°C ± 2°C, light/dark cycle: 12 h/12 h, humidity: 50% ± 10%). After 1 week of acclimatization, the rats were used to produce SQP-containing serum. The Animal Experiment Research Center of Zhejiang Chinese Medical University issued the experimental animal usage license SYXK (Zhejiang) 2021–0012. The study protocol was approved by the Institutional Animal Care and Use Committee of Zhejiang Chinese Medical University (ZSLL-2021–103).

#### 2.6.2 Animal grouping and administration

CKD models were established as previously described (Liu Y. et al., 2024). In the CKD model study, the control group (n = 9) was maintained under normal conditions. The adenine group (27 mice) was administered 75 mg/kg adenine for 14 days. According to clinical administration guidelines, the human dosage of SQP is 24 pills per dose, three times daily, with each pill weighing approximately 0.17 g, equating to ∼0.068 g/kg/day in adults. Using a body surface area normalization method (Nair and Jacob, 2016), the clinically equivalent dose for mice was calculated to be 0.8364 g/kg/day. Accordingly, two SQP treatment groups (n = 9 per group) received 0.8 or 1.6 g/kg/day SQP via oral gavage for 50 days (Xie et al., 2024; Huang et al., 2025). Distilled water was used to treat both the control and the leftover CKD model groups during the same period. On day 14, blood was collected from the orbits of randomly selected mice in the adenine and normal groups for biochemical analysis, followed by kidney dissection and pathological staining to assess the model. Urine was collected in metabolic cages to detect renal function indicators 2 h after the last administration on day 50. Subsequently, after a 12-h fasting period without water restriction, the mice were sedated with 3% pentobarbital sodium administered intraperitoneally, then dissected, and tissue samples were kept at −80°C.

To examine SQP as an intervention for OA, SQP-containing serum was produced according to previously described protocols (Liu Y. et al., 2024). Briefly, 18 SD rats were randomly assigned to three groups (n = 6). The SQP group was orally administered SQP (0.8 and 1.6 g/kg) for 5 days, whereas the control group received an equivalent volume of distilled water. The rats were fasted for 12 h prior to dissection. Two hours following the final intake, the rats were sedated with sodium pentobarbital (150 mg/kg), blood was drawn from the abdominal aorta, and serum was separated by centrifugation at 3000 rpm for 15 min at 4°C. Serum samples were heat-inactivated at 56°C for 30 min, filtered through a 0.22 µm filter, and preserved at −80°C for further analysis.

### 2.7 Biochemical analysis

Serum urea nitrogen (BUN), serum creatinine (Scr), and 24-h urine protein (24h-UTP) were measured using an automated biochemical analyzer (Hitachi 7020, Tokyo, Japan).

### 2.8 Histopathological observation of renal tissues

Mouse kidney tissues were fixed in 4% paraformaldehyde, embedded in paraffin, and sectioned into 4 µm coronal slices. Following the manufacturer’s instructions, sections were stained with hematoxylin and eosin (H&E) using an H&E staining kit (C0105S, Beyotime Biotechnology, shanghai) and with β-galactosidase *in situ* using a β-galactosidase staining kit (C0602, Beyotime Biotechnology, shanghai). Renal tissue sections were stained using a Masson’s trichrome staining kit (C0189S, Beyotime Biotechnology, Shanghai, China). Images were captured using a light microscope at ×400 magnification. TEM (Hitachi H-7650, Tokyo, Japan) was used to investigate and photograph the ultrastructures of the kidney.

### 2.9 RNA-seq analysis

Total RNA was isolated from renal tissues and maintained at −80°C by homogenizing them in Trizol reagent (Thermo Fisher, 15,596,018) following the manufacturer’s instructions. The amount and purity of total RNA were determined using a Bioanalyzer 2,100 and RNA 6000 Nano LabChip kit (Agilent, CA, USA, 5067–1511), and RNA samples with an RNA integrity number more than 7.0 were utilized to generate sequencing libraries. Five microliters of total RNA from each sample were reverse transcribed to create cDNA libraries, which were then paired-end sequenced on the Illumina NovaSeq platform. Hangzhou LianChuan Biotechnology Co., Ltd. performed the sequence-related experiments.

Sequencing data from the Illumina platform were processed using the DESeq2 software to detect differentially expressed genes (DEGs) between the SQP and model groups. The DAVID database was used to conduct KEGG pathway enrichment analyses.

### 2.10 ELISA assay

Take out a frozen kidney tissue sample and cut a piece (∼20 mg) for homogenization. The levels of IL-1β (lot. 70-EK201B), IL-6 (lot. 70-EK206) and TNF-α (lot. 70-EK282) in the kidney were determined using a mouse ELISA kit (LianKe, Hangzhou, China) according to the manufacturer’s instructions.

### 2.11 Cell culture

The mouse germ cell line (GC-1spg) was cultured as previously described (Duarte et al., 2024). Every 2–3 days, the culture medium was replaced, and the cells were passaged at 80%–90% confluence.

### 2.12 CCK8 analysis

To assess the safety of the SQP-containing serum, GC-1spg cells were seeded at a density of 3 × 10^3^ cells per well in a 96-well plate. The cells were grown for 24 h in a serum-free medium (SF), medium containing 10% FBS, medium containing 10% normal rat serum (NRS), and medium containing 10% SQP-containing serum (1.6 g/kg SQP). To perform the CCK8 examination, we added 10 µL of the reagent to each well and incubate them at 37°C for 2 h. A microplate reader was used to measure the optical density (OD) at 450 nm.

### 2.13 β-galactosidase staining

Take out the frozen kidney tissue samples, embed them with OCT and make frozen sections, and then stain the samples according to the instructions of the cell senescence β-galactosidase staining kit (C0602, Beyotime, Shanghai) after rewarming. For cells cultured in 6-well plates, aspirate the cell culture medium, wash once with PBS, and then stain the cells according to the instructions of the kit.

### 2.14 Western blot analysis

Total protein from kidney tissues and GC-1spg cells was extracted, separated by SDS-PAGE, and transferred to a PVDF membrane. The membrane was then blocked with 5% nonfat milk at room temperature for 1 h. Then, primary antibodies targeting β-tubulin (AC015, ABclonal), histone H3 (BF9211, Affinity), SIRT1 (BF0189, Affinity), NF-κB (BF5006, Affinity), IκBα (AF5002, Affinity), and p-IκBα (AF 2002, Affinity) were incubated at 4°C overnight. After washing, the membranes were incubated with a secondary antibody and examined using an Odyssey CLx imager (LI-COR Biosciences, Lincoln, Nebraska, USA). Image Studio v5.2 was employed to capture signals, and the results were normalized as fold changes relative to the β-tubulin and histone H3 expression levels.

### 2.15 Immunofluorescence staining

Kidney samples embedded in paraffin were sectioned into 5-μm-thick slices. Antigen retrieval was performed and endogenous peroxidase activity was blocked in paraffin-embedded liver sections. Subsequently, these sections were treated with SIRT1 (BF0189, Affinity) and NF-κB antibodies (BF5006, Affinity). After washing with PBS, the sections were incubated with Cy5-labeled goat anti-mouse IgG secondary antibodies (A10524; Invitrogen) and FITC-labeled goat anti-rabbit IgG (F-2761; Invitrogen). Following washing, DAPI counterstaining was performed and digital images were collected at ×200 magnification using a fluorescence microscope (Nikon, Tokyo, Japan).

### 2.16 Statistical analysis

The staining results were statistically analyzed using ImageJ v1. 53p. Statistical analyses were performed using Prism 8.0 (GraphPad Software, Inc.). Two-tailed unpaired *t*-tests were used for two-group comparisons, and one-way ANOVA was used for multiple-group comparisons. Statistical significance was set at P < 0.05. All data are presented as mean ± SD.

## 3 Results

### 3.1 UPLC-Q-TOF/MS results of the aqueous extract of SQP

**FIGURE 1:**
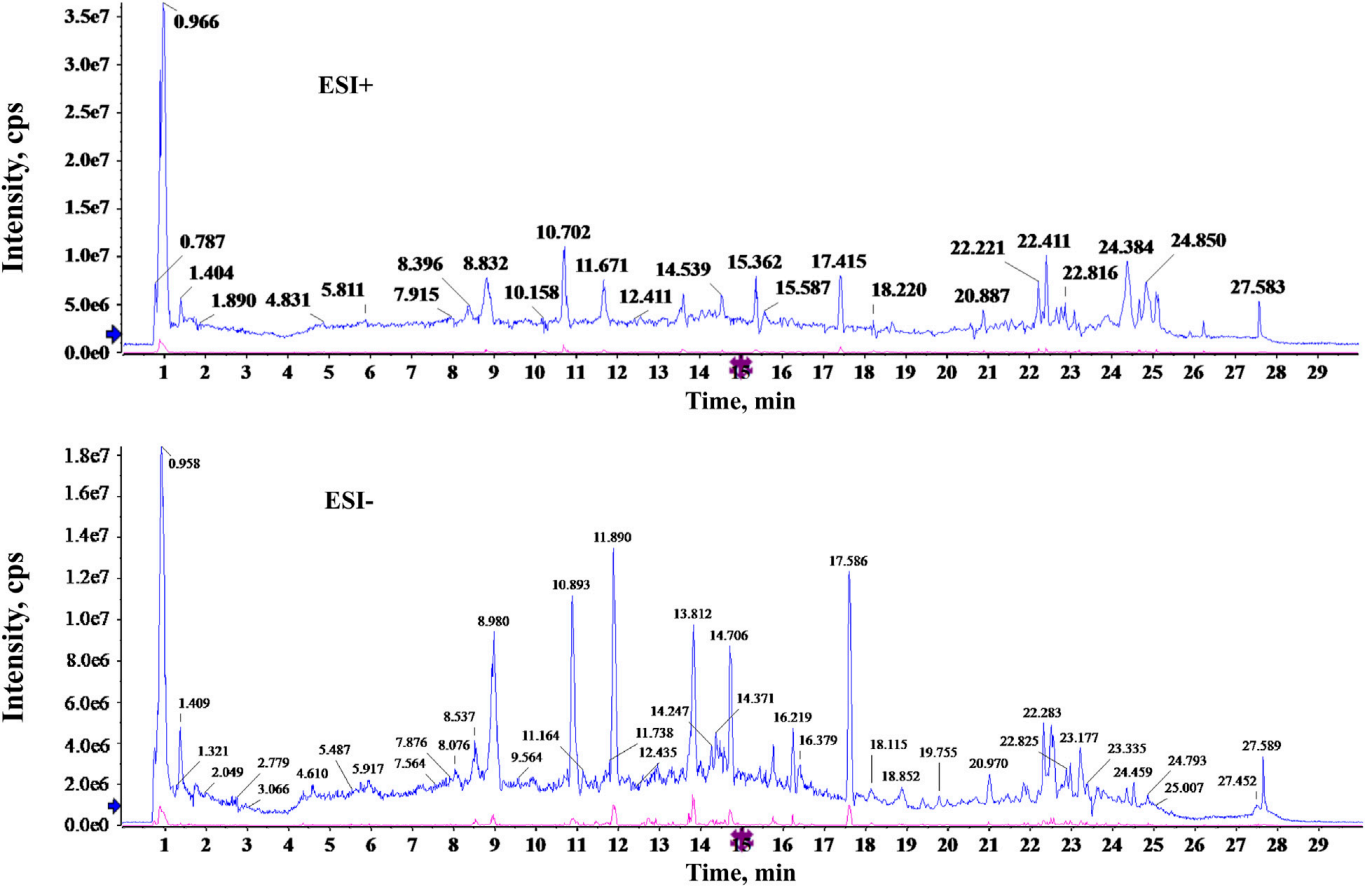
Total ion chromatogram of SQP water extract (A: positive ion mode, B: negative ion mode).

To identify the potentially active components of SQP, the major components of the aqueous extract were analyzed using UPLC-Q-TOF/MS. The total ion chromatogram revealed the detection of 32 compounds in both positive and negative ion modes (Figure 1); detailed component information is provided in Supplementary Table S2.

### 3.2 Predicted target networks of each herbal ingredient in SQP and disease target networks of CKD and OA

**FIGURE 2:**
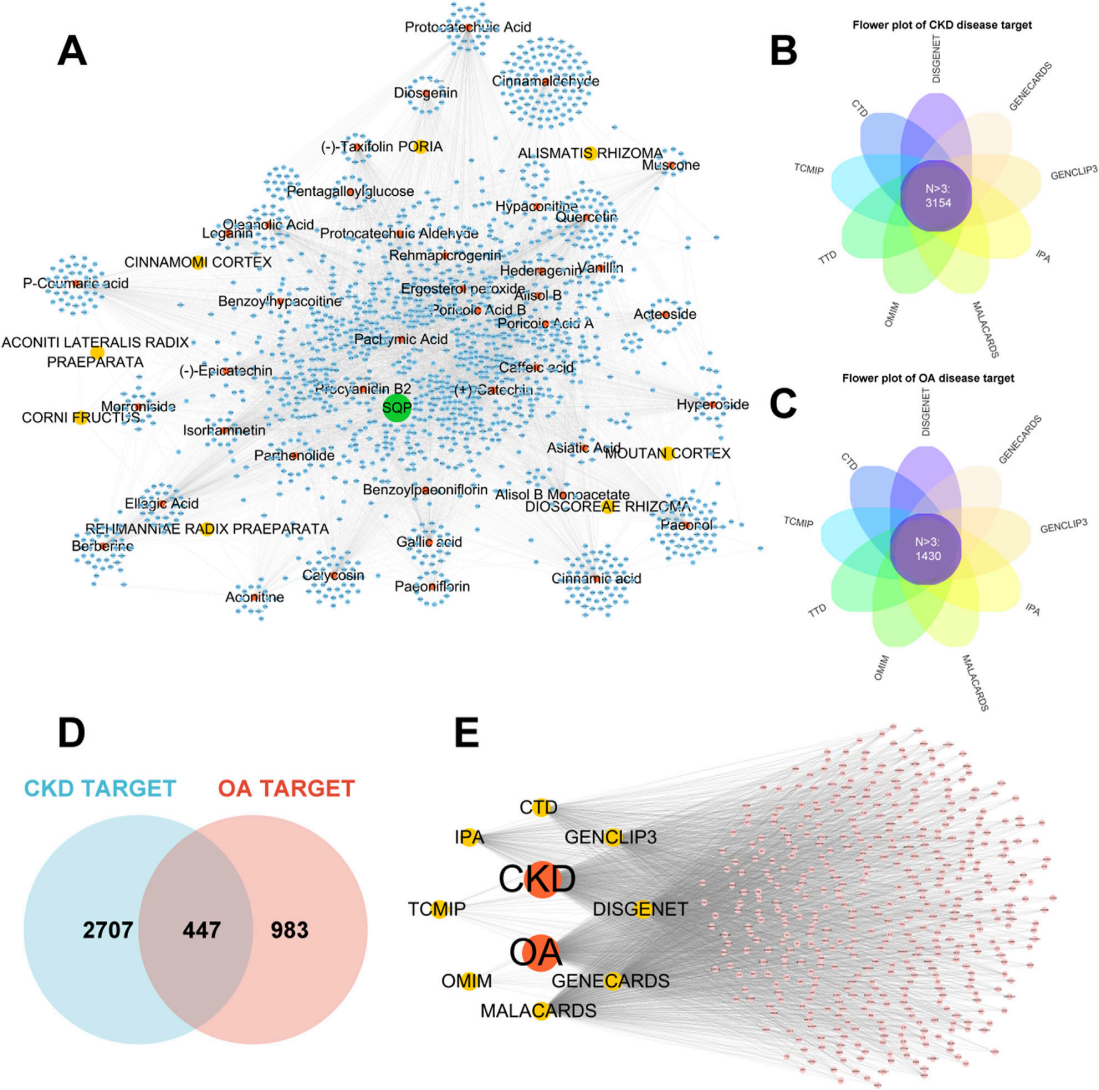
Predicted target networks of each herbal ingredient in SQP and disease target networks of CKD and OA. **(A)** Network graph of components of herb in SQP and theie prediction targets. **(B, C)** Flower plot of high frequency CKD and OA disease targets. **(D, E)** Venn diagram and network graph of the intersection targets between CKD and OA diseases.

In this study, 32 active components were identified using UPLC-Q-TOF-MS analysis. Literature review supplemented the identification of 10 active components in SQP: (−)-taxifolin, (+)-catechin, alisol B, alisol B monoacetate, diosgenin, ergosterol peroxide, hederagenin, hypaconitine, poricoic acid A, and quercetin. The target sites of 41 active components were predicted using multiple databases, resulting in 1,641 targets (Figure 2A). Using multi-source disease databases and Upset R package, 3,154 and 1,430 high-frequency targets (N > 3) were identified for CKD and OA, respectively (Figures 2B,C). By overlapping these disease targets, we finally identified 447 common disease targets of CKD and OA and mapped their network (Figures 2D,E).

### 3.3 Network pharmacology assessment of the mechanism of homotherapy for heteropathy in CKD and OA using SQP

**FIGURE 3:**
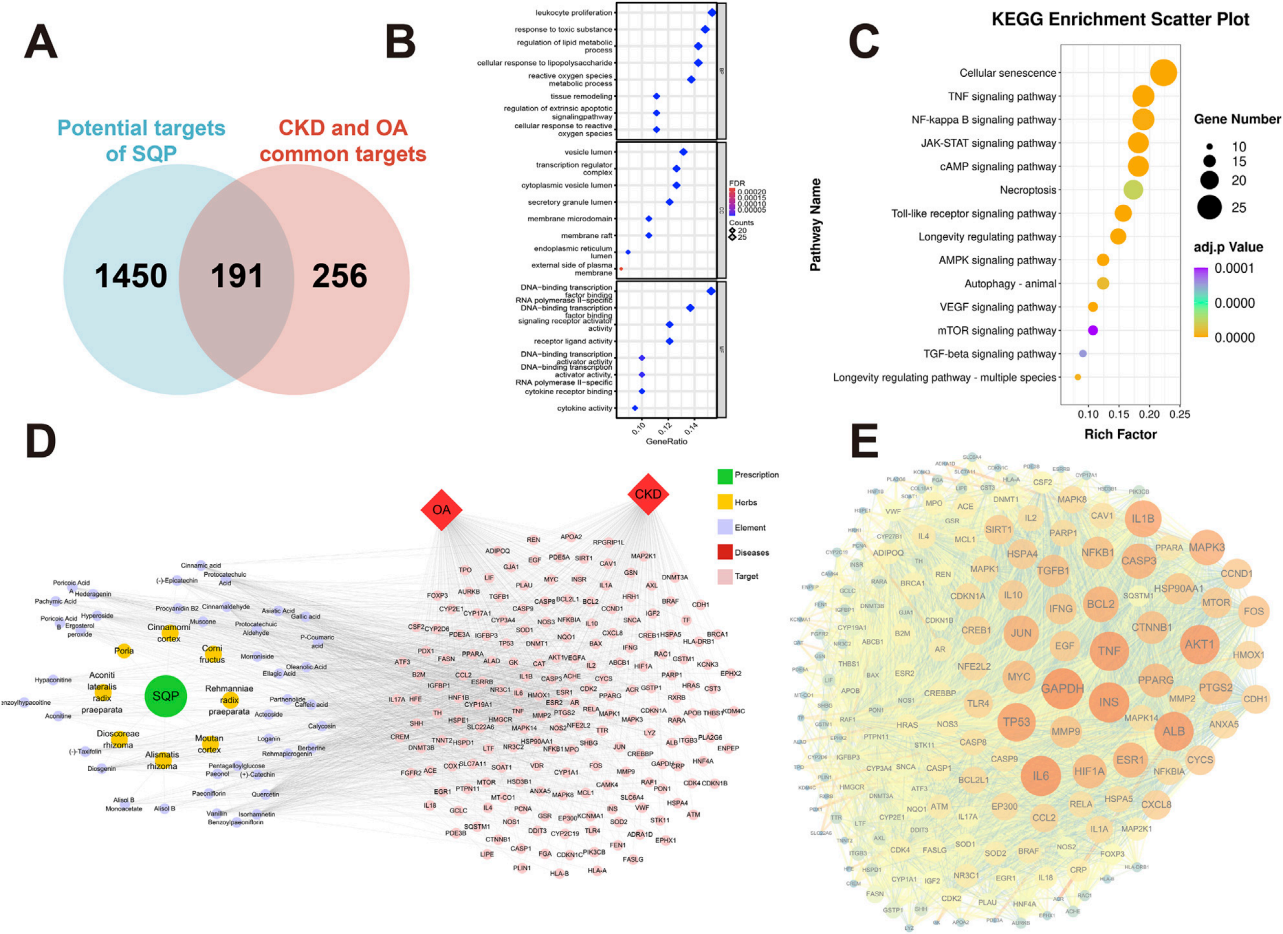
Network pharmacology investigation into the mechanism underpinning SQP-based homotherapy for heteropathy. **(A)** Venn diagram of the intersection of SQP component prediction targets with common targets for CKD and OA. **(B, C)** Bubble chart of GO **(B)** and KEGG **(C)** enrichment analyses. **(D)** Network graph of components of SQP, associated diseases, and their overlapped targets. **(E)** PPI network plot.

Further study of the molecular mechanism of SQP in the treatment of CKD and OA through network pharmacology. Intersectional analysis of the predicted components and disease targets revealed 191 common targets (Figure 3A). Functional enrichment analysis demonstrated that SQP exerts its effects by modulating biological processes such as the lipopolysaccharide response, cytokine production, and oxidative stress response. It regulates biological pathways including cellular senescence, HIF-1 signaling, TNF signaling, NF-κB signaling, and necroptosis (Figures 3B,C). The formulation-component-target-disease network was visualized (Figure 3D), and the PPI network analysis of the intersecting targets indicated that key targets such as SIRT1, TP53, RELA, JUN, NFκB1, TNF, IL-6, and IL-1β may play a crucial role in SQP’s homotherapy for heteropathy effects (Figure 3E).

### 3.4 Machine learning analysis of clinical datasets to screen key senescence targets in CKD and OA

**FIGURE 4:**
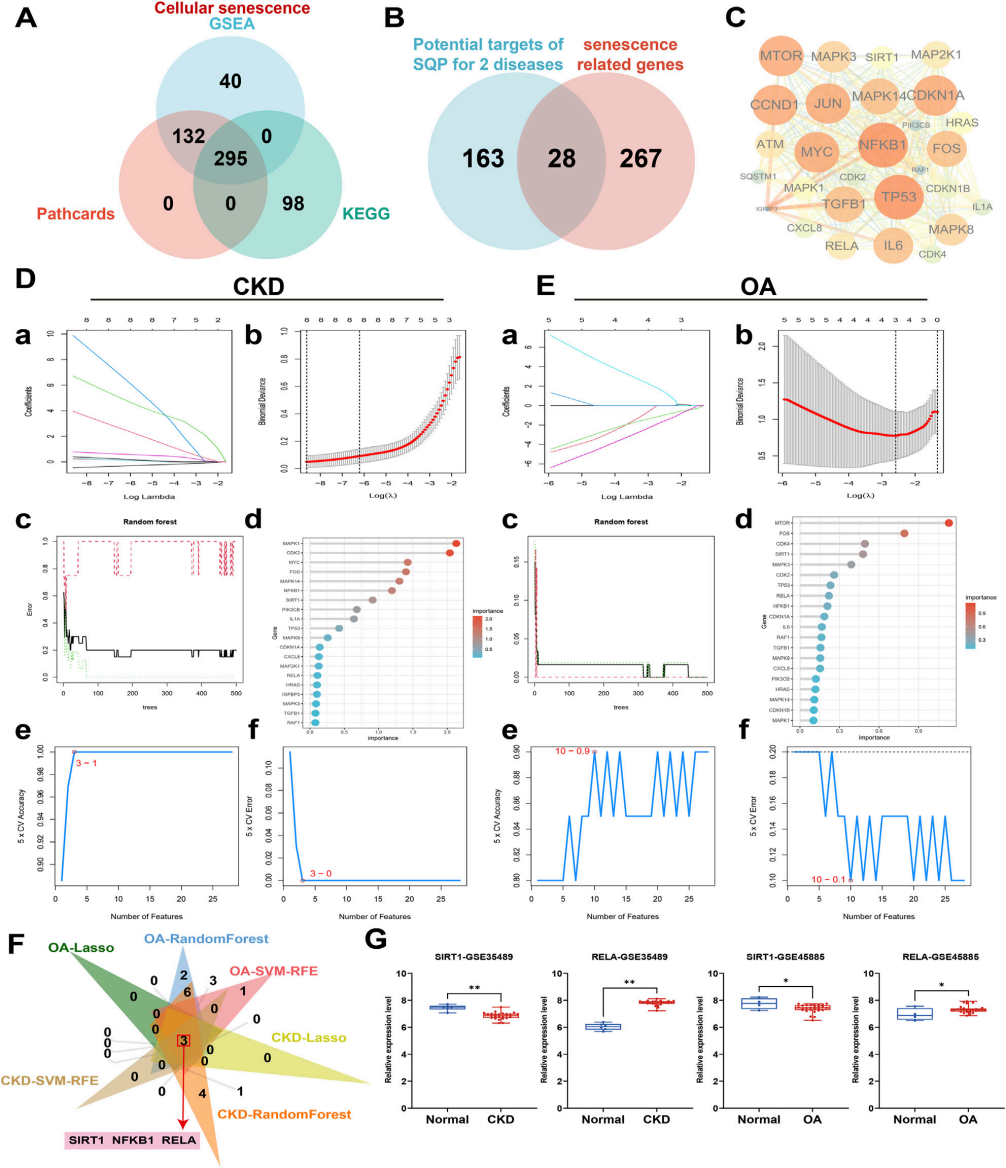
Machine learning analysis of clinical datasets to screen key senescence targets in CKD and OA. **(A)** Cell senescence gene background construction. **(B)** Overlap between the genetic background of cellular senescence and the potential targets of SQP for intervention in CKD and OA. **(C)** PPI network diagram of 28 senescence-related targets. **(D, E)** LASSO **(a, b)**, RandomForest, **(c, d)** and SVM-RFE **(e, f)** algorithm deployment for screening cell senescence targets related to the occurrence and development of CKD and OA. **(F)** Venn diagram of machine learning results. **(G)** Box plots of SIRT1 and RELA relative expression levels in other CKD and OA clinical datasets. Data are presented as mean ± SD. Compared with the normal group: *P < 0.05, **P < 0.01.

To investigate the role of the cellular senescence pathway in SQP’s alleviation of CKD and OA, a pathway background was constructed using databases and analyzed alongside network pharmacology results. A total of 295 shared cellular senescence targets were identified across three databases (Figure 4A), with 28 overlapping genes identified as potential targets of SQP intervention in CKD and OA (Figure 4B). A PPI network was constructed explored their interaction (Figure 4C). To further investigate the roles of these 28 cellular senescence-related genes in CKD and OA, three classical machine learning algorithms were applied to their clinical datasets to identify key targets. Based on the results of a binomial family model, LASSO regression identified eight essential features in the CKD-GSE66494 dataset (Figures 4D, a, b) and three in OA-GSE45887 (Figures 4E, a, b). The results from the RandomForest and SVM-RFE algorithms further revealed the ranking of genes, identifying those with the highest importance (Figures 4D, E, c–f). A Venn diagram of the machine learning results shows that the targets SIRT1, RELA, and NFKB1 may play a key role in the occurrence and development of CKD and OA (Figure 4F). SIRT1 and RELA has been further validated in other CKD and OA datasets (Figure 4G).

### 3.5 SQP alleviates adenine-induced renal injury

**FIGURE 5:**
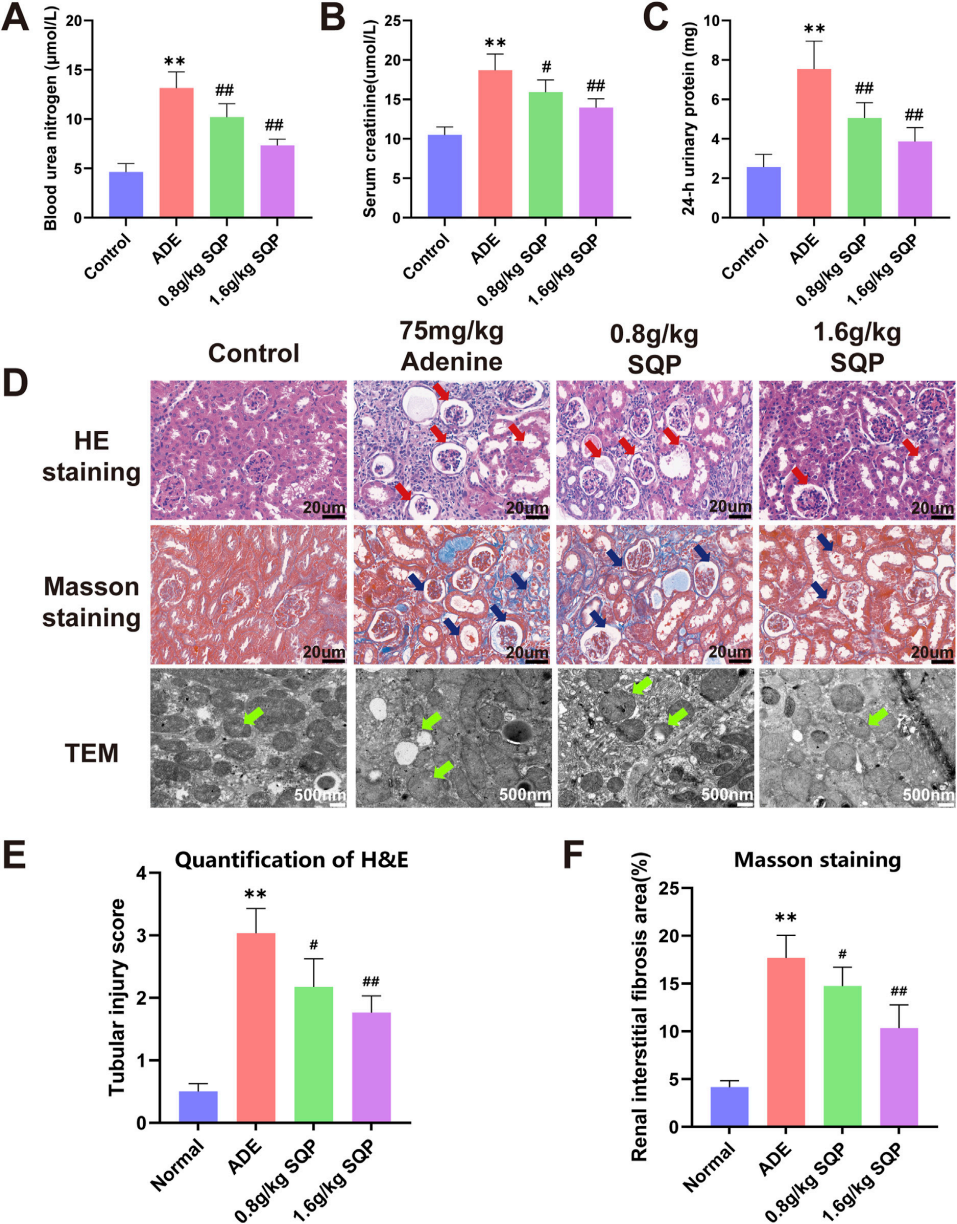
SQP alleviates adenine-induced renal injury. **(A–C)** The levels of BUN, Scr, and 24h U-TP. **(D)** H&E and Masson staining were performed to assess renal pathology (×400). Red arrows highlight areas of glomerular atrophy and tubular dilation in H&E staining, blue arrows indicate glomerular atrophy, tubular dilation, and collagen deposition in Masson staining, and green arrows denote changes in mitochondrial morphology. **(E, F)** Quantification of H&E staining and Masson staining positive area. Data are presented as mean ± SD (n = 9). Compared with the normal group: *P < 0.05, **P < 0.01. Compared with the model group: #*P* < 0.05, ##*P* < 0.01.

Biochemical and histopathological analyses on day 14 showed that the 75 mg/kg adenine group had significantly impaired renal function (**P < 0.05), with glomerular swelling, tubular injury, interstitial inflammation, and increased collagen deposition, indicating renal fibrosis. These findings confirm the successful establishment of a stable CKD model (Supplementary Figure S1). Biochemical analyses and pathological staining were performed to assess the effects of SQP on adenine-induced kidney damage. Biochemical indicators of renal function showed that 75 mg/kg adenine significantly increased the levels of BUN, 24 h U-TP, and Scr levels in CKD mice (Figures 5A–C, *P < 0.05, **P < 0.01), whereas SQP (0.8 and 1.6 g/kg) significantly reversed these renal dysfunctions (#*P* < 0.05, ##*P* < 0.01, Figures 5A–C). Histopathological and TEM analyses revealed that the renal tissue in the normal group was well-preserved, with glomeruli and renal tubules exhibiting regular morphology. Mitochondria displayed intact structure and margins, with no signs of rupture or fusion (Figures 5D–F). In contrast, the adenine model group showed necrosis, edema, vacuolar degeneration of renal tubular epithelial cells, significant inflammatory infiltration, tubular dilation, atrophy, and thickening of some glomerular basement membranes. Mitochondria were swollen, with disorganized cristae (Figures 5D–F, *P < 0.05, **P < 0.01). Following SQP intervention, inflammatory cell infiltration was significantly reduced, tubular dilation and atrophy were notably improved, and mitochondrial abnormalities were alleviated (Figures 5D–F, #*P* < 0.05, ##*P* < 0.01). These results indicate that SQP significantly ameliorated adenine-induced renal injury in mice.

### 3.6 RNA-seq analysis reveals that SQP may alleviate CKD by modulating cellular senescence and the NF-κB pathway

**FIGURE 6:**
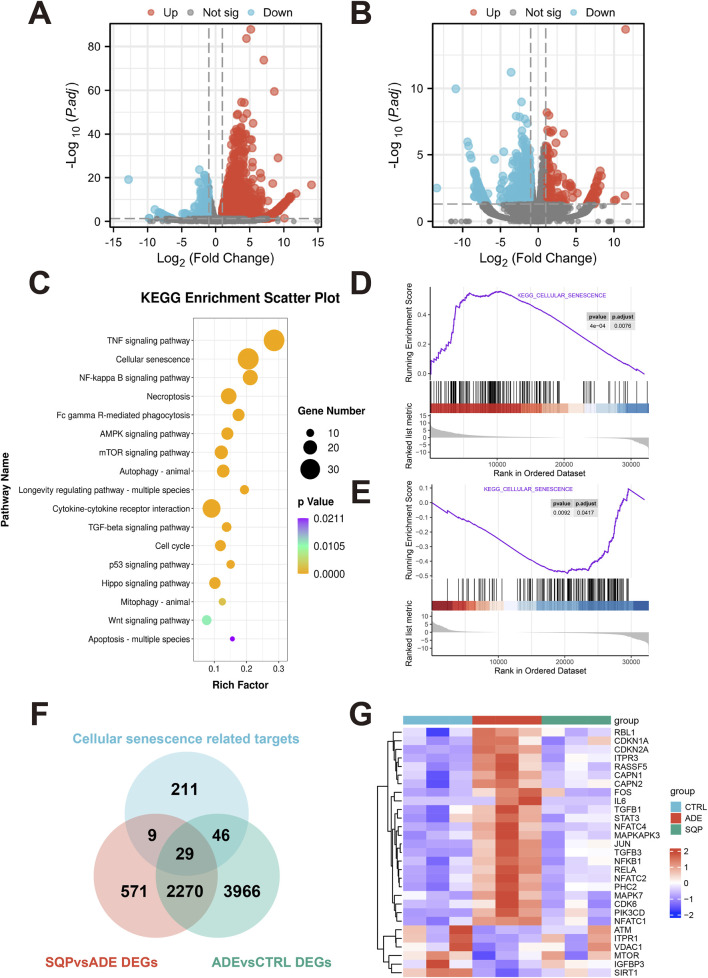
RNA-Seq analysis reveals that SQP may alleviate CKD by modulating cellular senescence and the NF-κB pathway. **(A, B)** Volcano plot of DEGs in the ADE vs. CTRL group **(A)** and the SQP vs. ADE groups **(B)**. **(C)** Bubble chart of KEGG pathway enrichment analysis of DEGs in the SQP vs. ADE group. **(D, E)** GSEA curve of cellular senescence pathway in the ADE vs. CTRL group **(D)** and the SQP vs. ADE groups **(E)**. **(F)** Venn diagram of the overlap of RNA-Seq DEGs and cellular senescence-related genes. **(G)** Heatmap of overlapping DEGs expression level in RNA-Seq.

To further investigate the molecular mechanisms by which SQP alleviates adenine-induced renal injury, RNA-Seq analysis was performed on kidney tissue samples. DEGs were identified using an adjusted *P* < 0.05 and |log2FoldChange| > 0.58, comparing the adenine-treated group with the wild-type group (ADE vs. CTRL, Figure 6A) and the 1.6 g/kg SQP intervention group with the adenine-treated group (SQP-H vs. ADE, Figure 6B). KEGG enrichment analysis indicated that SQP may alleviate renal injury by affecting pathways such as cellular senescence, the NF-κB signaling pathway, and the AMPK signaling pathway (Figure 6C). GSEA analysis results showed that the cellular senescence pathway might be activated in the ADE group (Figure 6D), and SQP could significantly improve this performance (Figure 6E). Subsequently, we extracted the DEGs in the context of cellular senescence in RNA-Seq (Figure 6F) and analyzed their expression levels. The heatmap showed that ADE significantly activated the expression of cellular senescence-related genes, while SQP improved this situation (Figure 6G). These findings suggest that SQP may alleviate CKD by attenuating cellular senescence pathway.

### 3.7 SQP mitigate senescence-associated secretory phenotype (SASP) relase and cellular senescence in CKD

**FIGURE 7:**
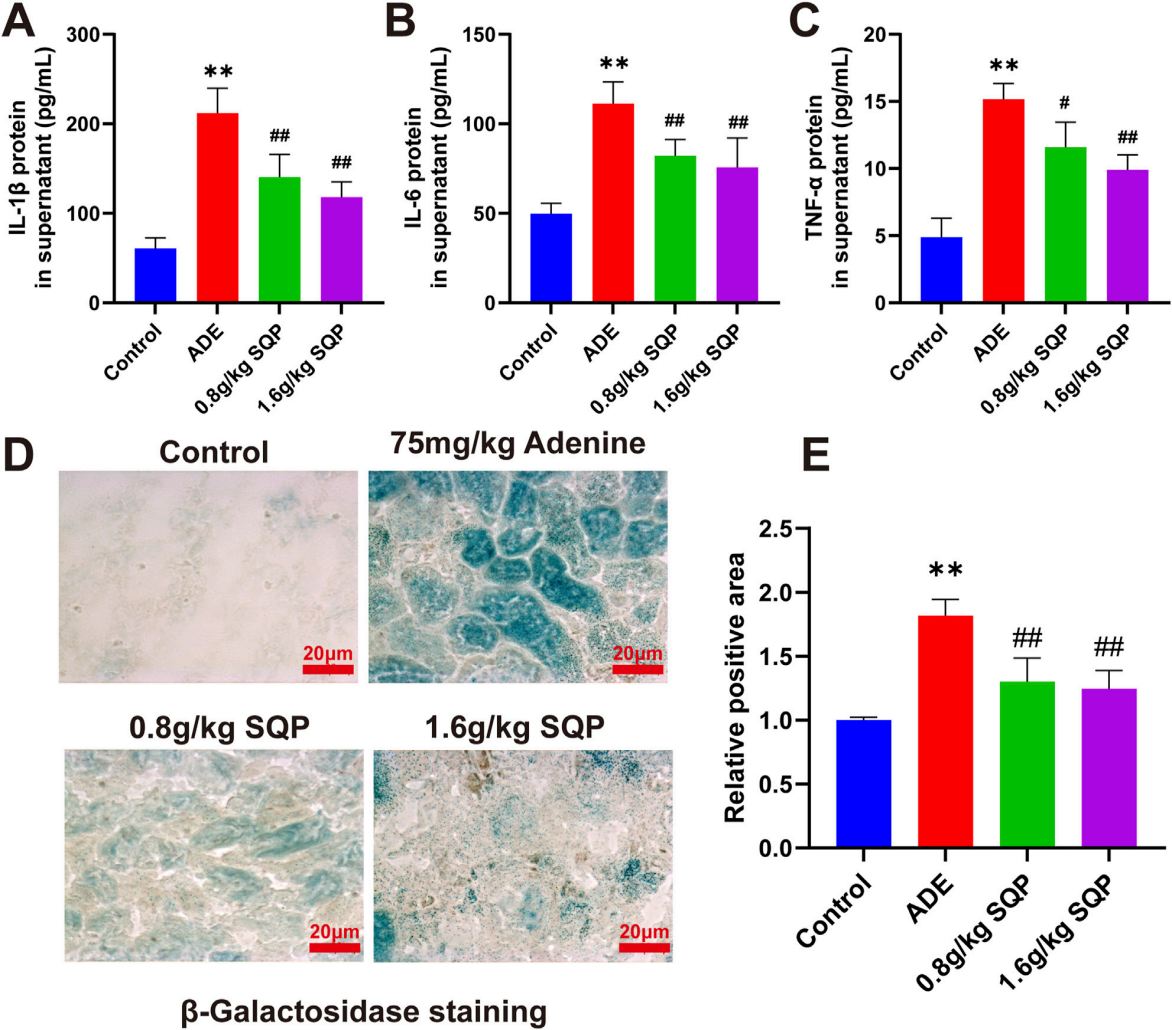
Impact of SQP on SASP release and cellular senescence in CKD. **(A–C)** Concentration of IL-1β, IL-6 and TNF-α in tissue supernatant. **(D, E)** Results and statistical analysis of β-galactosidase staining in renal tissue. Data are presented as mean ± SD (n = 3); compared with the normal group, *P < 0.05, **P < 0.01; compared with the model group, #*P* < 0.05, ##*P* < 0.01.

To investigate the effect of SQP on SASP release in adenine-induced CKD. The results showed that, compared to the control group, the levels of IL-1β, IL-6, and TNF-α were significantly increased in the model group. However, these cytokines were significantly reduced in the SQP-treated groups compared to the model group, with the high-dose SQP group exhibiting the most pronounced effects (Figures 7A–C). We then used β-galactosidase labeling to explore whether SQP modifies cellular senescence in CKD mouse renal tissue. The results demonstrated a considerable increase in the positive area in the adenine-treated group, whereas SQP (0.8 and 1.6 g/kg) significantly ameliorated tubular epithelial cell senescence (Figures 7D,E, *P < 0.05, **P < 0.01). These findings indicate that SQP may mitigate CKD by reducing the release of SASP and inhibiting cellular senescence.

### 3.8 SQP modulates the SIRT1/NF-κB pathway to mitigate cellular senescence and alleviate CKD

**FIGURE 8:**
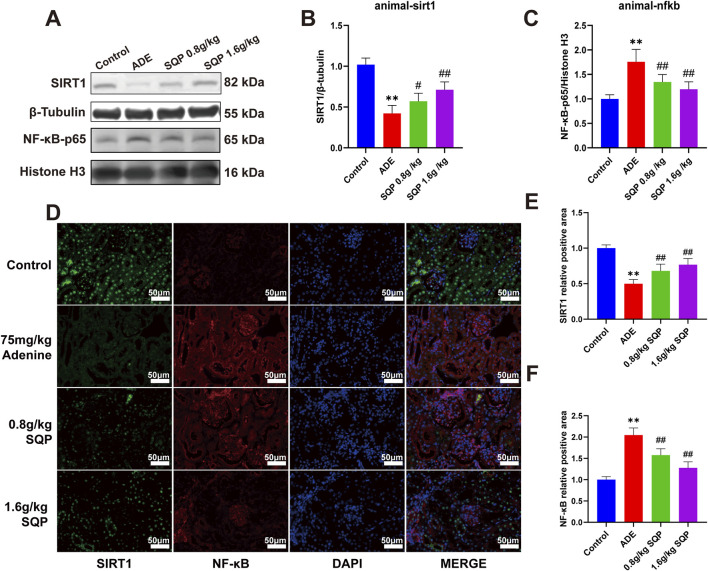
Impact of SQP on SIRT1/NF-κB pathway in CKD. **(A)** Western blots representative bands. **(B, C)** Quantification of SIRT1 and NF-κB protein levels in renal. **(D–F)** Immunofluorescence staining and statistical analysis of SIRT1 and NF-κB in mouse kidney tissue. Data are presented as mean ± SD (n = 3); compared with the normal group, *P < 0.05, **P < 0.01; compared with the model group, #*P* < 0.05, ##*P* < 0.01.

We then assessed protein expression in the SIRT1/NF-κB pathway in renal tissue to better understand SQP’s mechanism (Figure 8A). Western blot analysis revealed that adenine treatment dramatically reduced SIRT1 expression and enhanced NF-κB nuclear translocation (Figures 8B,C, *P < 0.05, **P < 0.01). On the contrary, SQP intervention prevented NF-κB from translocation to the nucleus and dramatically increased SIRT1 expression. (Figures 8B,C, #*P* < 0.05, ##*P* < 0.01). Immunofluorescence staining further confirmed these results (Figures 8D–F). These findings suggest that SQP may alleviate CKD by attenuating cellular senescence via regulating the SIRT1/NF-κB pathway.

### 3.9 SQP improves D-galactose-induced spermatogonial cell senescence by modulating the SIRT1/NF-κB pathway

**FIGURE 9:**
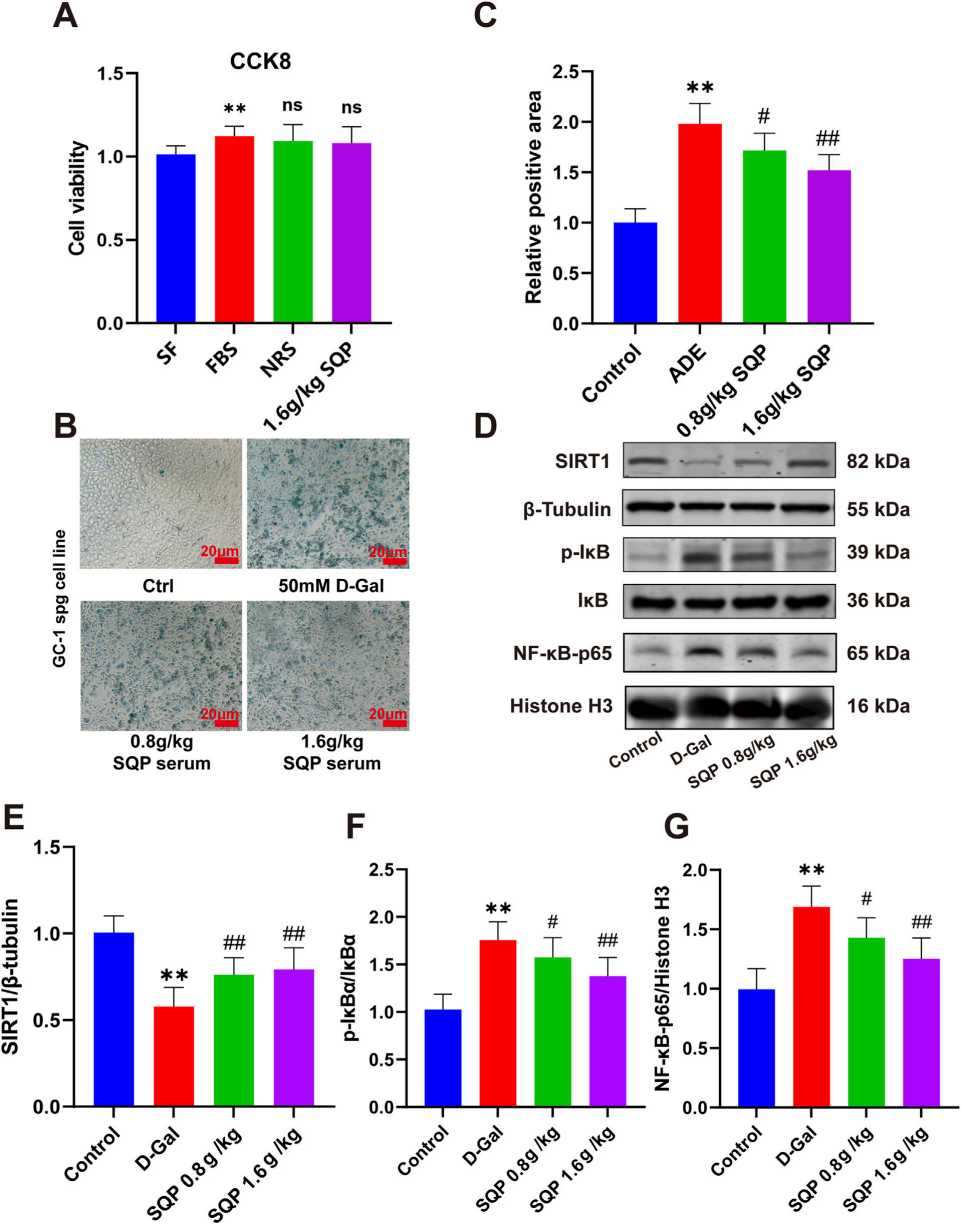
Impact of SQP on cellular senescence and the SIRT1/NF-κB pathway in GC-1spg cells. **(A)** CCK8 assay results. **(B, C)** Results and statistical analysis of β-galactosidase staining in GC-1spg cells. **(D)** Western blots representative bands. **(E–G)** SIRT1 and NF-κB expression in GC-1spg cells determined by western blots. Data are presented as mean ± SD (n = 3); compared with the normal group, *P < 0.05, **P < 0.01; compared with the model group, #*P* < 0.05, ##*P* < 0.01.

In the following investigation, a classical OA cell model was established by administering D-galactose, followed by assessing and the safety of SQP-containing serum using the CCK8 assay. The results showed that DMEM containing 10% NRS and 10% SQP-containing serum had no significant effect on cell viability (Figure 9A, *P* > 0.05). β-galactosidase staining analysis indicated that exposure to 50 mM D-galactose for 24 h induced senescence in GC-1spg cells (Figures 9B,C, *P < 0.05, **P < 0.01), whereas SQP-containing serum significantly alleviated cell senescence in a dose-dependent manner (Figures 9B,C, #*P* < 0.05, ##*P* < 0.01). Western blot analysis was performed to explore the effect of SQP on the SIRT1/NF-κB pathway in GC-1spg cells (Figure 9D). The results revealed that D-galactose markedly reduced SIRT1 expression, raised the p-IκBα/IκBα ratio, and promoted NF-κB nuclear translocation (Figures 9E–G, *P < 0.05, **P < 0.01). However, SQP-containing serum significantly restored SIRT1 expression, inhibited the p-IκBα/IκBα ratio, and reduced NF-κB nuclear expression (Figures 9E–G, #*P* < 0.05, ##*P* < 0.01). These results suggest that SQP-containing serum ameliorates cellular senescence induced OA by modulating the SIRT1/NF-κB pathway.

## 4 Discussion

The incidence of CKD and its associated complications increase annually, making it a significant contributor to the global disease burden and severely affecting patients’ quality of life (Collaboration, 2020). Currently, the clinical drugs used to treat CKD and its complications include renin-angiotensin-aldosterone system (RAAS) drugs, hypoglycemic agents, mineralocorticoid receptor antagonists, and erythropoiesis-stimulating agents (Huang et al., 2023). However, the risks and benefits of these drugs require further evaluation (Laville et al., 2024), highlighting the urgent need for complementary and alternative therapies with improved efficacy and fewer side effects in clinical practice. Moreover, effective medications for treating OA are limited, with current approaches mainly focusing on lifestyle changes (such as quitting smoking and alcohol). These approaches include antioxidant therapy, endocrine therapy, and TCM (Zafar et al., 2023; Lin et al., 2024).

It is noteworthy that TCM considers that CKD and OA share common characteristics, with their pathogenesis attributed to “kidney yang deficiency” (Wang et al., 2022; Gao et al., 2023). According to TCM, CKD falls under categories such as “edema,” “urinary retention,” and “kidney fatigue” with kidney deficiency being a central aspect throughout the disease (Wang et al., 2022). In TCM, OA is categorized under “cold semen,” “infertility,” and “oligospermia”. TCM posits that the kidney is the foundation of innateendowment, storing essence and governing reproduction; both kidney yin and yang are considered essential in spermatogenesis (Wang J. et al., 2021). Kidney yang deficiency is a common syndrome in patients with CKD and OA, making tonifying the kidney yang a frequently employed therapeutic strategy. Clinical studies have demonstrated that wenshenyang decoction can significantly improve renal function in CKD patients with kidney yang deficiency, with a favorable safety profile (Jin et al., 2025). Liu et al. reported that huangqi guizhi wuwu decoction alleviates podocyte injury and ameliorates IgA nephropathy by modulating the AT1R/Nephrin/c-Abl signaling pathway (Liu et al., 2021). Yao et al. and Ma et al. further revealed that icariin improves DKD-associated renal fibrosis and OA symptoms through activation of the AR/RKIP and ERα/c-Fos signaling pathways, respectively (Ma X. et al., 2024; Yao et al., 2024). Moreover, Guilingji was shown to elevate serum glucose-6-phosphate levels, enhance mitochondrial function and energy metabolism, mitigate testicular damage associated with kidney yang deficiency, and improve sperm quality (Feng et al., 2024). Moreover, Guilingji was shown to elevate serum glucose-6-phosphate levels, enhance mitochondrial function and energy metabolism, mitigate testicular damage associated with kidney yang deficiency, and improve sperm quality (Feng et al., 2024). In recent years, numerous studies have found that increased levels of oxidative stress and inflammatory markers are frequently present during the onset and progression of CKD, affecting male sperm quality and increasing the risk of OA (Zhang et al., 2017; Barkabi-Zanjani et al., 2020; Tsao et al., 2020).

Differentiation and therapy based on syndrome patterns constitute the foundation of TCM as well as the primary concept driving illness diagnosis and treatment. Based on this principle, the concept of “homotherapy for heteropathy” has emerged, which posits that different diseases with shared pathogenesis can be treated with the same or similar therapeutic approaches (Ding et al., 2023). Clinical studies have shown that the pathogenesis of these two diseases is related to the kidney yang deficiency syndrome. As a classic formula for warming the kidney and tonifying yang, SQP has shown significant “homotherapy for heteropathy” effects on these diseases, although the mechanisms remain unclear.

Network pharmacology, well-known for its capacity to handle large volumes of nonlinear and complicated data, enables the efficient and systematic explanation of possible interactions between the multi-components of TCM and disease targets. This method has emerged as a frontier and hotspot in TCM research. Recent research has indicated that network pharmacology offers a unique perspective and approach to understanding the processes behind the “homotherapy for heteropathy” occurrence in TCM. This technique can discover the shared mechanisms of action and underlying molecular foundations by building network links between medication components, targets, pathways, and illnesses (Wang X. et al., 2021). Liu et al. used network pharmacology to investigate the mechanisms of steamed *Panax notoginseng* in “homotherapy for heteropathy” for anemia and Alzheimer’s disease and found that it exerted its therapeutic effects by reducing the expression of TNF-α and TLR4 in anemic rats and transgenic Aβ flies (Mengnan et al., 2023). In this study, network pharmacology results suggested that shenqi pills may regulate biological processes such as immune activation and oxidative stress, regulate cell senescence, TNF signaling pathway and NF-κB signaling pathway, and play a role in improving CKD and OA.

Machine learning algorithms have emerged as a significant research tool in the field of biomedicine, with numerous recent studies employing these methods to investigate disease biomarkers (Bachelot et al., 2023; Liu P. et al., 2024). Dashtban et al. applied seven machine learning algorithms to population-based electronic health records (2006–2020), identifying five CKD subtypes with distinct internal validity, prognostic outcomes, and medication use. These findings offer new insights into CKD etiology, treatment, and risk prediction (Dashtban et al., 2023). Zeadna et al. conducted a retrospective cohort study involving 119 patients who underwent testicular sperm extraction (TESE) at an IVF center between 1995 and 2017. Using machine learning algorithms, they developed a regression model that evaluates the probability of TESE success based on parameters such as FSH, LH, and testosterone levels. This model aids in addressing male infertility caused by non-obstructive azoospermia (NOA) (Zeadna et al., 2020). To explore the molecular mechanism of SQP in alliviating CKD and OA, we utilized network pharmacology, RNA-Seq, machine learning, and both *in vivo* and *in vitro* studies. Our research indicates that SQP may intervene in CKD and OA primarily by inhibiting SIRT1/NF-κB pathway-mediated cellular senescence.

The SASP is a hallmark of cellular senescence, characterized by the secretion of bioactive molecules such as proinflammatory cytokines, chemokines, matrix remodeling proteases (MMPs), and growth factors (Maus et al., 2023). Recent studies have underscored the pivotal role of SASP in the progression of CKD and OA (Mylonas et al., 2021; Zhang W. et al., 2023). Key SASP components, including IL-1β, IL-6, and TNF-α, are particularly implicated in these conditions. For instance, Chen et al. reported elevated IL-6 levels in renal fibrosis associated with diabetic nephropathy (Chen et al., 2022), while Ijima et al. observed persistent increases in IL-1β, IL-6, and TNF-α in mouse models of lupus nephritis (Ijima et al., 2022). Additionally, Ayusso et al. demonstrated that green tea extract mitigates drug-induced renal injury by regulating SASP markers such as IL-1β and IL-6 (Ayusso et al., 2024).Recent studies have found that abnormal activation of myeloid fibroblasts promotes the synthesis of extracellular matrix (ECM), cell senescence of renal tubular epithelial cells, macrophage polarization and abnormal activation of the cGAS-STING pathway exacerbate the chronic inflammatory microenvironment and aggravate the process of fibrosis (An et al., 2022; Ma Y. et al., 2024; Jiao et al., 2025).

In male infertility, aging within the testicular somatic cell microenvironment is a key contributing factor (Alfano et al., 2021). He et al. confirmed through single-cell RNA sequencing that SASP expression is markedly elevated in reproductive-related cells, such as Leydig cells, in older individuals (He et al., 2024). Moreover, active compounds from natural products in classical prescriptions have been shown to improve male infertility by modulating SASP markers, including IL-1β, IL-6, and TNF-α(Li et al., 2022; Zhang X. et al., 2023; Ma K. et al., 2024). The above studies confirm that SASP plays a key role in the progression of CKD and OA. Our findings reveal that Shenqi Pills significantly reduce SASP secretion and cellular senescence induced by adenine. Furthermore, serum containing Shenqi Pills markedly alleviates senescence in mouse spermatocytes triggered by D-gal.

Mammalian sirtuins are NAD-dependent deacetylases that play vital roles in the regulation of cellular metabolism, oxidative stress, and inflammatory responses, of which SIRT1 is the most extensively studied (Wu et al., 2022). In recent years, studies have reported that SIRT1 not only interacts with JMJD3 to inhibit macrophage polarization and SASP production, exerting mitochondrial protection and anti-renal fibrosis effects, but also interacts with TEAD1 to improve mitochondrial function and alleviate kidney injury. These findings suggest that SIRT1 may serve as a key upstream target that exerts anti-fibrotic effects through multiple coordinated pathways (Seok et al., 2018; An et al., 2023; Tran et al., 2025).

Numerous studies conducted in the last few years have demonstrated SIRT1’s crucial function in aging-related disorders. These studies highight the importance of SIRT1 activation in alleviating the progression of CKD and OA and emphasize SIRT1’s central role in maintaining homeostasis and regulating metabolism (Li et al., 2019; Dhillon et al., 2024). Recent research has found that CKD leads to decreased SIRT1 expression in the glomeruli and increased expression of senescence and inflammation markers. The *Atractylodes lancea* and *Magnolia officinalis* combination alleviates tubular epithelial cell senescence and reduces proteinuria and renal fibrosis by restoring SIRT1 activity (Feng et al., 2021; Yang et al., 2023). Furthermore, sperm from *SIRT1*-knockout mice are susceptible to oxidative stress associated damage, exhibiting morphological abnormalities, increased apoptosis, and DNA damage, while resveratrol can improve sperm quality and embryonic development by activating SIRT1 (Tatone et al., 2018). Our study demonstrated that SQP treatment significantly restored SIRT1 expression and delayed cellular senescence, thereby positively impacting CKD and OA.

NF-κB is a fundamental nucleus transcription element that manipulates inflammation. Recent studies have revealed its close association with the development and progression of CKD and OA (Chen et al., 2012; Zhang J. Q. et al., 2023). Multiple studies have demonstrated that SIRT1 interacts with RelA/p65, a subunit of NF-κB, thereby inhibiting the senescence and inflammatory responses caused by NF-κB signaling (Nopparat et al., 2017; Sun et al., 2021). Recent findings indicate that inhibiting NF-κB signaling within renal tubular epithelial cells can reduce inflammation and renal fibrosis caused by senescence, which may be a potential strategy for treating CKD (O'Sullivan et al., 2023; Wang et al., 2024). Kim et al. showed that Korean ginseng inhibits the release of cytokines related to inflammation and senescence in the kidneys of aged mice by suppressing NF-κB signaling (Kim et al., 2021). The inflammatory microenvironment induced by senescence can disrupt spermatogenesis, leading to reduced sperm counts, decreased sperm quality, and even azoospermia (Akbari et al., 2022; Ozawa et al., 2023; Zhang W. et al., 2023). Li et al. showed that the Bazi Bushen formula alleviates the decline in sperm quality in aged mice by inhibiting the NF-κB-mediated inflammatory response (Li et al., 2021). Lu et al. discovered that the natural product icariin can reduce testicular inflammation, alleviate apoptosis, and prevent blood-testis barrier damage by inhibiting NF-κB nuclear translocation (Lu et al., 2024). Building on this, our study found that SQP effectively alleviates inflammatory responses in CKD and OA by inhibiting NF-κB nuclear translocation.

Numerous studies have identified SIRT1 as a key upstream molecule in the NF-κB signaling pathway, where activation of SIRT1 effectively inhibits the stimulation of the NF-κB pathway, thus alleviating cellular senescence (Nopparat et al., 2017; Sun et al., 2021). Recent studies have shown that SIRT1 alleviates renal inflammation and fibrosis by inhibiting the nuclear translocation of NF-κB, reducing the expression of proinflammatory factors (TNF-α, IL-6, IL-1β) and chemokines (MCP-1) (Yan et al., 2022); at the same time, it activates antioxidant enzymes (SOD, CAT), inhibits the TGF-β/Smad pathway and mitochondrial damage, and reduces oxidative stress and cell senescence (Tatone et al., 2018; Wu et al., 2024). The overactivation of NF-κB caused by the blockage of SIRT1 promotes the generation of ROS, forming an oxidative stress-inflammation positive feedback loop, exacerbating renal damage, testicular spermatogenesis disorders, and sperm DNA damage (Suleiman et al., 2020; Yuan et al., 2022).Previous research has shown that treatment with the SIRT1 agonist BF175 in CKD mice significantly attenuates NF-κB activation and the expression of senescence and inflammatory markers, thereby reducing albuminuria and the progression of CKD (Feng et al., 2021). Additionally, Cui et al. found that quercetin reduces cellular senescence, NF-κB levels, and damage caused by oxidation in the liver and kidney tissues of a diabetic mouse model by upregulating SIRT1 activity (Cui et al., 2022).

Compounds such as α-lipoamide (Tan et al., 2025), dapagliflozin (Wang H. et al., 2025), Zuogui decoction (Xie et al., 2025), 6′-O-caffeoylarbutin (Wang Y. et al., 2025), and mulberry extract (Adthapanyawanich et al., 2025) exert protective effects by upregulating SIRT1 and its downstream targets (e.g., PPARγ, p53), suppressing NF-κB signaling, reducing proinflammatory cytokines (TNF-α, IL-1β, IL-6), enhancing antioxidant enzyme activities (SOD, CAT), and improving mitochondrial function. These findings highlight the therapeutic potential of targeting SIRT1/NF-κB signaling in cellular senescence-related and metabolic diseases.

**FIGURE 10:**
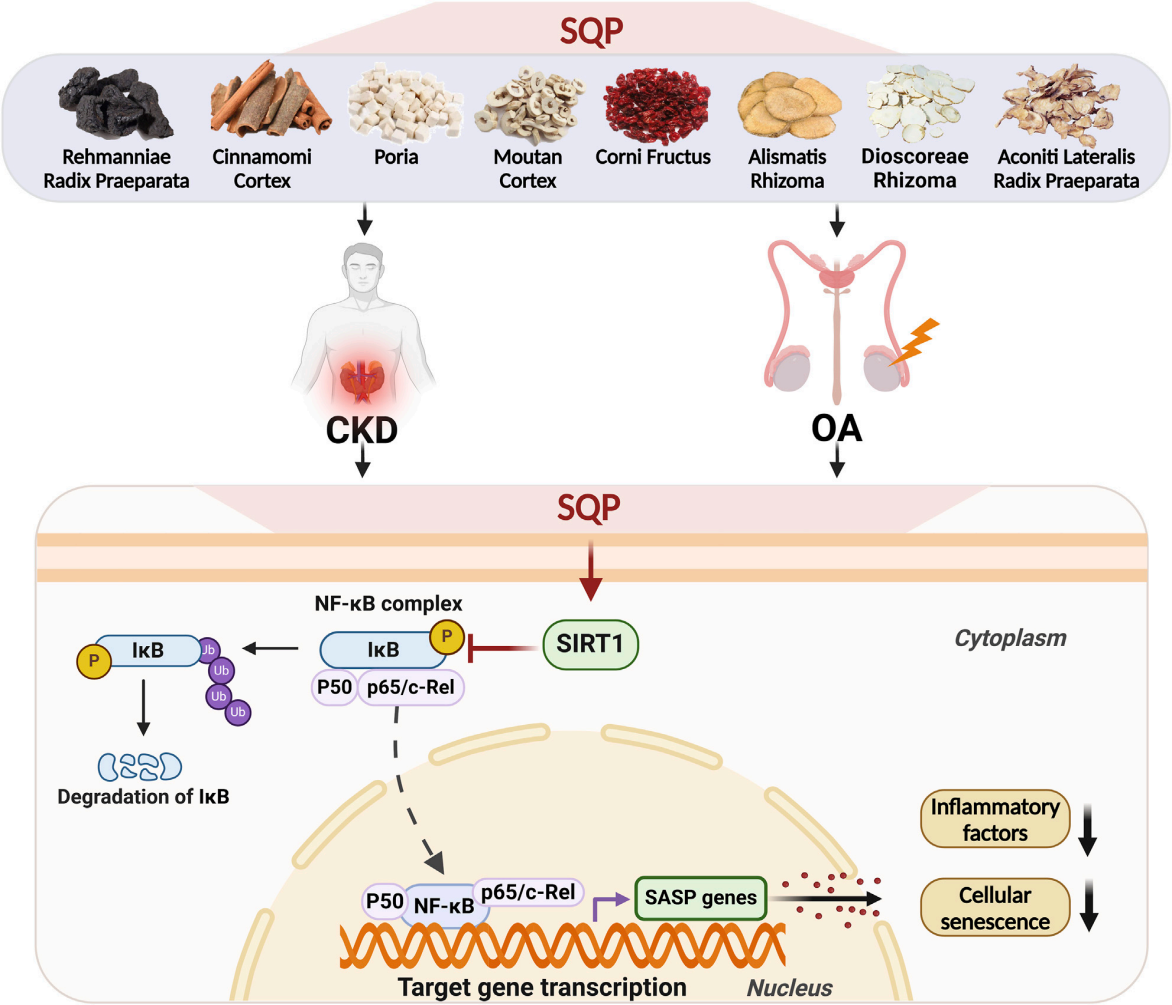
Schematic illustration of the mechanism by which SQP regulates the SIRT1/NF-κB pathway to alleviate cellular senescence and treat CKD and OA.

Our results indicate that SQP treatment significantly restores SIRT1 expression, inhibits NF-κB nuclear translocation, and reduces the release of inflammatory factors, thereby reversing cellular senescence. Based on this evidence, we demonstrated that SQP may inhibit cellular senescence and senescence-associated inflammation by regulating the SIRT1/NF-κB pathway, and effectively treating CKD and OA using the homotherapeutic approach (Figure 10).

## 5 Conclusion

This study systematically elucidated the mechanisms of action of SQP in the treatment of CKD and OA using a unified therapeutic approach through a research strategy combining network pharmacology, RNA-seq, and *in vivo* and *in vitro* experiments. Overall, SQP may prevent aging and inflammation by activating SIRT1, inhibiting NF-κB translocation, and reducing SASP release (Figure 9). This could serve as the foundation and supports the TCM view of SQP’s effectiveness in “homotherapy for heteropathy” in CKD and OA, all of which share a common kidney-yang deficiency syndrome. This finding provides an innovative perspective for understanding the therapeutic effects of SQP on these diseases.

## Data Availability

The data presented in the study are deposited in the GEO repository (https://www.ncbi.nlm.nih.gov/geo/), accession number GSE66494, GSE35489, GSE45885 and GSE45887.

## Abbreviations

SQP, shenqi pill; CKD, chronic kidney disease; OA, oligoasthenospermia; TCM, traditional Chinese medicine; LPS, Lipopolysaccharide; UPLC-Q/TOF-MS, ultra-high performance liquid chromatography with quadrupole time-of-flight mass spectrometry; GFR, glomerular filtration rate; ESRD, end-stage renal disease; ESI, electrospray ionization; IDA, information-dependent acquisition; DP, declustering potential; PPI, protein-protein interaction; SPF, specific-pathogen free grade; BUN, Serum urea nitrogen; Scr, serum creatinine, 24h-UTP, 24 h urine protein; H&E, hematoxylin and eosin; GO, gene ontology; KEGG, kyoto encyclopedia of genes and genomes; LASSO, least absolute shrinkage and selection operator; SVM-RFE, support vector machine - recursive feature elimination; SF, serum-free medium; NRS, normal rat serum; SIRT1, Sirtuin 1; NF-κB, nuclear factor kappa-light-chain-enhancer of activated B cells; IκBα, NF-Kappa-B Inhibitor Alpha; p-IκBα, Phospho NF-Kappa-B Inhibitor Alpha; SASP, senescence-associated secretory phenotype; IL-1β, interleukin 1 beta; IL-6, interleukin 6; TNF-α, tumor necrosis factor α.
