# Supplementary material for: Homotherapy for heteropathy of chronic kidney disease and oligoasthenozoospermia through regulating SIRT1/NF-κB pathway by Shenqi pills
Source: Front Pharmacol. 2025 Jun 9;16:1551423. doi: 10.3389/fphar.2025.1551423 (PMC12183175; doi:10.3389/fphar.2025.1551423)
Supplement: Supplementary file 1 [file Supplementaryfile1.docx]

After oral administration for 14 consecutive days, the mice in the 75mg/kg adenine model group were blooded from their orbits, and the supernatant was centrifuged for biochemical detection. The samples were then dissected and the kidneys were pathologically analyzed. The results showed that compared with the normal group, the renal function of the mice in the model group was significantly impaired (** P<0.05). HE staining showed that the renal tissue structure of the normal group was clear, the glomeruli were regular in morphology, the tubular epithelial cells were closely arranged, and there were no abnormalities such as inflammation or necrosis. In the 75mg/kg adenine model group, the renal tissue showed glomerular swelling, nuclear abnormalities, degeneration and shedding of tubular epithelial cells, and obvious cell infiltration in the renal interstitium, indicating tissue damage and inflammatory response. The results of Masson staining showed that the collagen fibers in the normal group were sparsely and evenly distributed, without obvious fibrosis. In the 75mg/kg adenine model group, the collagen fibers in the renal tissue increased significantly, especially concentrated in the glomeruli and renal interstitium, indicating the presence of renal fibrosis.


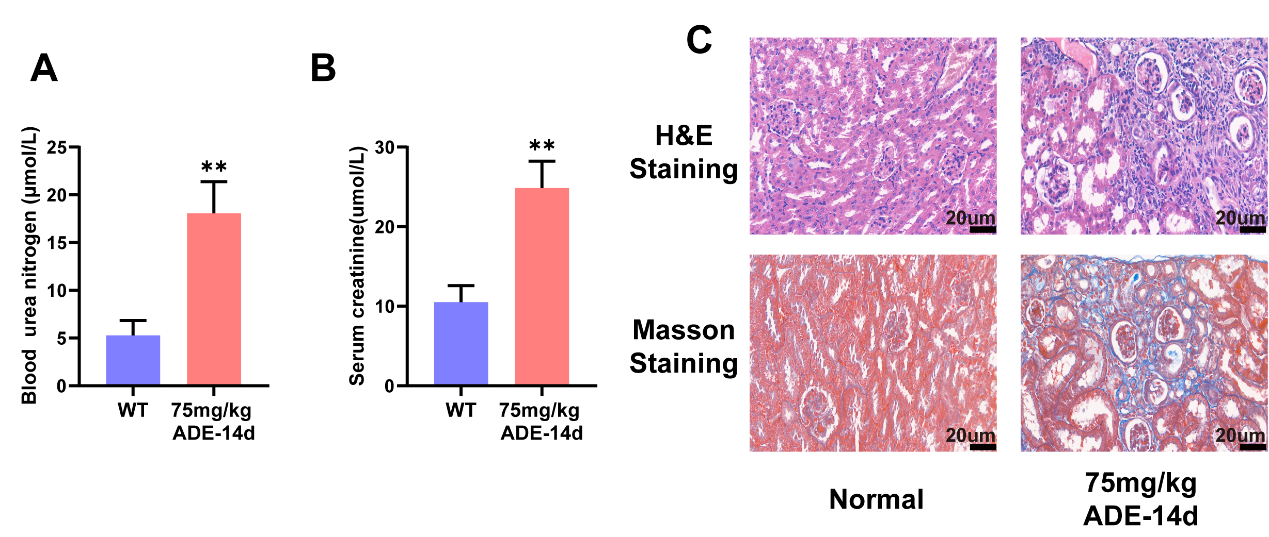


Supplementary Figure 1. Model evaluation of mice in the oral administration of 75 mg/kg adenine model group on day 14. (A-B) Analysis of blood urea nitrogen and serum creatinine in mice. (C) H&E and Masson staining were performed to assess renal pathology (×400). Data are presented as mean ± SD (n = 9). Compared with the normal group: * *P* < 0.05, ** *P* < 0.01.
